# The New War Loan

**Published:** 1898-07

**Authors:** L. J. Gage

**Affiliations:** Secretary; Treasury Department, Office of the Secretary, Washington, D. C.


### THE NEW WAR LOAN.

TREASURY DEPARTMENT, OFFICE OF THE SECRETARY,
WASHINGTON, D. C., June 13th, 1898.

The Secretary of the Treasury invites subscriptions from the people of the United States for $200,000,000 of the bonds of the 3 per cent. loan authorized by the act of Congress to provide ways and means to meet war expenditures. Subscriptions will be received at par for a period of thirty-two days, the subscription being open from this date to three o'clock P. M. on the 14th day of July, 1898. The bonds will be issued in both coupon and registered form, the coupon bonds in denominations of $20, $100, $500, and $1,000, and the registered bonds in denominations of $20, $100, $500, $1,000, $5,000, and $10,000. They will be dated August 1st, 1898, and, by their terms, will be redeemable in coin at the pleasure of the United States after ten years from the date of their issue, and due and payable August 1st, 1918.

The bonds will bear interest at the rate of 3 per cent. per annum, payable quarterly; the interest on the coupon bonds will be paid by means of coupons, to be detached from the bonds as the interest becomes due, and the interest on the registered bonds will be paid by checks drawn to the order of the payees, and mailed to their addresses.

The law authorizing this issue of bonds provides that in allotting said bonds the several subscriptions of individuals shall be first accepted, and the subscriptions of the lowest amounts shall be first allotted. In accordance with that provision allotments to all individual subscribers will be made before any bonds will be allotted to other than individuals. All individual subscriptions for $500 or less will be allotted in full as they are received, and such subscriptions must be paid in full at the time the subscription is made. If the total sum subscribed for in amounts of $500 or less should exceed $200,000,000 the allotments will be made according to priority of the receipt of the subscriptions.

Allotments on subscriptions for over $500 will not be made until after the subscription closes, July 14th, and will then be made inversely according to the size of the subscription, the smallest subscription being first allotted, then the next in size next, and so on, preference being given to individual subscriptions. Persons subscribing for more than $500 must send in cash or certified checks to the amount of 2 per cent. of the sum subscribed for, such deposit to constitute a partial payment, and to be forfeited to the United States in the event of failure on the subscriber's part to make full payment for his subscription, according to the terms of the circular. Allotments to subscribers for more than $500 will be made as soon as possible after the subscription closes.

In order to avoid a too rapid absorption of funds into the Treasury, with a possible consequent evil effect on industry and commerce, any subscriber for more than $500 will be permitted to take his allotment of bonds in instalments of 20 per cent., taking the first instalment within ten days after the notice of the allotment, and the balance at four equal intervals of forty days each, in four instalments, each of 20 per cent. of the bonds allotted. Delivery of bonds will be made in instalments as payment for them is received, and payment must in all cases be made in full as the bonds are taken. The 2 per cent. deposit will apply on the final instalment. Any subscriber may pay for the whole amount allotted him within ten days from the date of the notice of his allotment. Interest will be adjusted from the time of the actual payment, whether paid in one sum or in instalments as permitted. Separate subscriptions from one individual, although made from time to time, will be aggregated and considered as one subscription for this issue of bonds.

The Secretary of the Treasury will receive in payment for the bonds post-office money orders payable at Washington, D. C., and checks, bank drafts, and express money orders collectible in the cities of New York, Boston, Philadelphia. Baltimore, Washington, Cincinnati, Chicago, St. Louis, New Orleans, and San Francisco. All money orders and bank drafts must be drawn in favor of the Treasurer of the United States. The money orders and bank checks so received will be forwarded for collection by the Department, and as soon as returns are obtained the subscriber will be credited with the amount of his subscription as of the date of collection. The Secretary will also receive in payment for the bonds certificates of deposit issued by the Assistant Treasurers of the United States in the above-named cities. These certificates of deposit may be obtained from any Assistant Treasurer in exchange for gold coin, gold certificates, standard silver dollars, silver certificates, United States notes, Treasury notes of 1890, and national bank notes; and the subscriber will be credited with the amount of his subscription as of the date of the certificate of deposit. The Secretary will also receive currency sent by registered mail or by express direct to the Treasury Department.

For the mutual convenience of the subscribers and the Department, a blank form of letter to accompany remittances has been prepared, and it may be obtained at the offices of national and State banks generally, at the several sub-treasuries of the United States, at any money-order post-office, and at any express office.

The bonds will be dated August 1st, 1898, and they will be forwarded to subscribers at the address designated by them free of expense for transportation as soon after that date as possible. The bonds will be accompanied by a check for the amount of interest due the subscriber at the rate of 3 per cent. from the date of his payment to August 1st, 1898.

All remittances and other communications relative to this loan should be addressed to the Secretary of the Treasury, Division of Loans and Currency, Washington, D. C.

All subscriptions must be received at the Treasury Department, Washington, D. C., not later than 3 o'clock P. M., Thursday, July 14th, 1898. No subscriptions received after that date and hour will be considered.

L. J. GAGE,
*Secretary.*

